# A modified percutaneous transforaminal endoscopic surgery for central calcified thoracic disc herniation at the T11/T12 level using foraminoplasty and decompression: A case report

**DOI:** 10.3389/fsurg.2023.1084485

**Published:** 2023-05-09

**Authors:** Hou Lisheng, Tian Suhuai, Zhang Dong, Zhou Qing

**Affiliations:** ^1^ Senior Department of Orthopedics, the Fourth Medical Center of PLA General Hospital, Beijing, China; ^2^ Department of Orthopedics, Anci District Hospital, Langfang, China

**Keywords:** modified PTES (percutaneous transforaminal endoscopic surgery), central calcified thoracic disc herniation, flexible endoscopic power drill, fluoroscopic foraminoplasty, endoscopic foraminoplasty, minimally invasive decompression, case report

## Abstract

**Background:**

Thoracic disc herniation (TDH) is uncommon. Central calcified TDH (CCTDH) is even rare. Traditional open surgery was considered a gold standard to treat CCTDH, but it was accompanied by a high risk of complications. Recently, a technique called percutaneous transforaminal endoscopic decompression (PTED) was adopted to treat TDH. Gu et al. designed a simplified PTED technique and named it percutaneous transforaminal endoscopic surgery (PTES) to treat various types of lumbar disc herniation; it offered the advantages of simple orientation, easy puncture, reduced steps, and little x-ray exposure. However, PTES to treat CCTDH has not been reported in the literature.

**Methods:**

Here, we describe the case of a patient with CCTDH treated with a modified PTES through the unilateral posterolateral approach under local anesthesia and conscious sedation by using a flexible power diamond drill. First, we report that the patient was treated with PTES with later-stage endoscopic foraminoplasty, with an inside-out technique employed at the initial endoscopic decompression stage.

**Results:**

A 50-year-old male with progressive gait disturbance and bilateral leg rigidity with paresis and numbness was diagnosed with CCTDH at the T11/T12 level on MRI and CT examinations. A modified PTES was performed on November 22, 2019. The total mJOA (modified Japanese Orthopedic Association) score preoperatively was 12. The method of the determination of incision and the soft tissue trajectory establishment process were the same as those in the original PTES technique. The foraminoplasty process was divided into initial fluoroscopic and final endoscopic stages. At the fluoroscopic stage, the hand trephine's saw teeth were just rotated into the lateral portion of the ventral bone from the superior articular process (SAP) to seize the SAP firmly, while at the endoscopic stage, in order to remove the ventral bone from the SAP safely under direct endoscopic visualization, adequate foramen enlargement was achieved without causing any risk of damage to the neural structures in the spinal canal. During the endoscopic decompression process, the soft disc fragments ventral to the calcified shell were undermined to form a cavity using an inside-out technique. Then, a flexible endoscopic diamond burr was introduced to degrade the calcified shell, and a curved dissector or a flexible radiofrequency probe was used to dissect the thin bony shell from the dural sac. Eventually, the shell was fractured within the cavity piece by piece to remove the whole CCTDH and achieve adequate dural sac decompression, resulting in minimal blood loss and no complications. The symptoms were gradually alleviated and the patient almost completely recovered at the 3-month follow-up, with no symptom recurrence found at the 2-year follow-up. The mJOA score improved to 17 at the 3-month follow-up and to 18 at the 2-year follow-up compared with 12 points preoperatively.

**Conclusions:**

A modified PTES may be an alternative minimally invasive technique for the treatment of CCTDH and provide similar or better outcomes over traditional open surgery. However, this procedure requires good endoscopic experience on the part of the surgeon and is beset with technical challenges and therefore should be performed with utmost care.

## Introduction

Thoracic disc herniation (TDH) is uncommon, accounting for 0.25%–0.75% of all disc herniations (1). Surgical treatment is indicated for those suffering from myelopathy and/or intractable neurological deficit (2), which constitutes approximately 0.15%–1.8% of all surgically treated disc herniations (3). Due to the anatomical characteristics of the thoracic spine, conventional open TDH surgery comes with great challenges and significant hazards with regard to a possible surgical access , which may result in serious complications (4). TDH may be soft or calcified (5). Calcified TDH (CTDH) is seen in approximately 40% of the TDH cases, and central CTDH (CCTDH) is even rare (6). An anterior or anterolateral transthoracic approach may be essential for CCTDH because it provides an excellent exposure of calcified discs with minimal or no manipulation of the dural sac (7, 8). However, these approaches are associated with significant morbidity and mortality rates relating to intercostal neuralgia, postoperative atelectasis, pneumothorax, pneumonia, and severe post-thoracotomy pain (9). A posterolateral approach is seldom performed for CCTDH because of inadequate ventral exposure in traditional open surgery. However, with the emergence and progress of minimally invasive spinal surgery (MISS), especially with the emergence and development of endoscopic spine surgery (ESS), a posterolateral approach has become acceptable (3, 10).

Smith initiated the ‘real’ MISS in 1963 by injecting chymopapain intradiscally, and this process was called ‘chemonucleolysis' (11). Encouraged by Smith's results, Kambin in 1970 initiated a feasibility study of mechanical nuclear debulking by inserting a Craig cannula *via* a posterolateral approach. In 1975, Hijikata inserted a cannula transforaminally into the disc space under the C-arm and successfully removed the nucleus pulposus, and this procedure was termed percutaneous nucleotomy (PN) (12). These minimally invasive access studies to the disc were rare (13). In 1997, Yeung designed the Yeung Endoscopic Spine System (YESS), a decompression procedure under direct endoscopic visualization, signaling the formal beginning of the modern era of ESS (14). This procedure was named “percutaneous endoscopic discectomy (PED)” by Japanese surgeons (10). Later in Europe, this procedure was further developed and was renamed “full-endoscopic discectomy (FED)” (15). The traditional approach in the PED technique was transforaminal, which we called PTED (percutaneous transforaminal endoscopic decompression); the other is the interlaminar approach (10). The transforaminal endoscopic spine system (TESSYS) technique advocated by Hoogland et al. made the PTED technique possible to operate the intracanal by foraminoplasty to enlarge the intervertebral foramen with special reamers (16). There are also other techniques in FED or PED, while YESS and TESSYS are considered by most as representing different concepts of FED: intradisc (inside-out) and intracanal (outside-in). Recently, some surgeons suggested to name the procedure “FED” to replace “PED or PTED” since the term “percutaneous” may cause confusion in that the procedure is similar to the C-arm-based non-endoscopic one (10, 17). But to date, “percutaneous” is used frequently in the ESS field, and it may take some more time for FED to gain complete acceptance (18, 19). FED or PED techniques differ from traditional arthroscopes in that they have an additional working channel port to allow instruments to be passed under direct endoscopic visualization with continuous saline irrigation (20).

YESS is initially centered around the lumbar regions for very limited disc herniations. Since the emergence of TESSYS, PTED evolved from an intradiscal procedure to a true foraminal epidural procedure through which both a targeted discectomy and a foraminal decompression can be performed. Nearly all kinds of lumbar disc herniations are accessible by TESSYS with the outside-in technique (18), but the complexity of the C-arm guiding orientation, the difficulty in finding the optimal trajectory for the target, and the large number of steps involved in surgical manipulation all lead to a greater exposure of x-ray, longer duration of the operation, and a steep learning curve (18).With advances in surgical techniques and technology in recent years, ESS techniques have been utilized for determining pathology in the cervical, thoracic, and lumbar spines (20).

Endoscopic transpedicular thoracic discectomy was first reported in 1999 (21). Recently, PTED was introduced to treat noncalcified TDH (1) and lateralized CTDH (22), and endoscope-assisted spinal surgery was found acceptable to treat CTDH (4). In 2017, Gu et al. reported a new PTED technique called “PTES” (percutaneous transforaminal endoscopic surgery) to treat lumbar disc herniation (LDH), which offered the advantages of simple orientation, easy puncture, reduced steps, and little x-ray exposure (18). In 2016, Wagner et al. reported about the use of PTED to treat TDH by employing a straight electric burr (23). In 2019, Liu et al. reported about the use of PTED to deal with a migrated TDH by employing an endoscopic reamer in the procedure of foramino-laminaplasty (24). Zhang et al. reported about the use of PTED to treat CTDH through bilateral approaches by employing a novel T rigid bendable burr (25). Houra and Saftic reported about the use of PTED for two-level CTDHs with straight and angled electric burrs for bone removal but with cannulated TomShidi needles to establish a decompression trajectory (26).

We adopted the PTES technique of Gu et al. to deal with lumbar disc herniations since early 2018 and made some modifications later that year with encouraging results. Then, we expanded the indications to deal with a lateralized soft TDH, again meeting with success. In this study, we present the case of a patient with CCTDH at the T11/12 level who underwent a unilateral modified PTES with a combination of endoscopic foraminoplasty using a hand trephine at the later foraminoplasty stage and a flexible endoscopic power diamond drill in the decompression process, resulting in a positive outcome.

## Case report

A 50-year-old male presented to our department on October 25, 2019, with a complaint of progressive gait disturbance and bilateral leg rigidity with paresis and numbness for 2 months. The onset of symptoms was insidious and developed progressively. No history of obvious trauma was recalled. A neurological examination showed a motor deficit of the bilateral legs (muscle strength: grade 3/5 for the left leg and grade 4/5 for the right leg) and an impaired superficial and deep sensation below the L1 dermatome. Patellar and ankle clonus and Babinski's signs were bilaterally positive.

**Figure 1:**
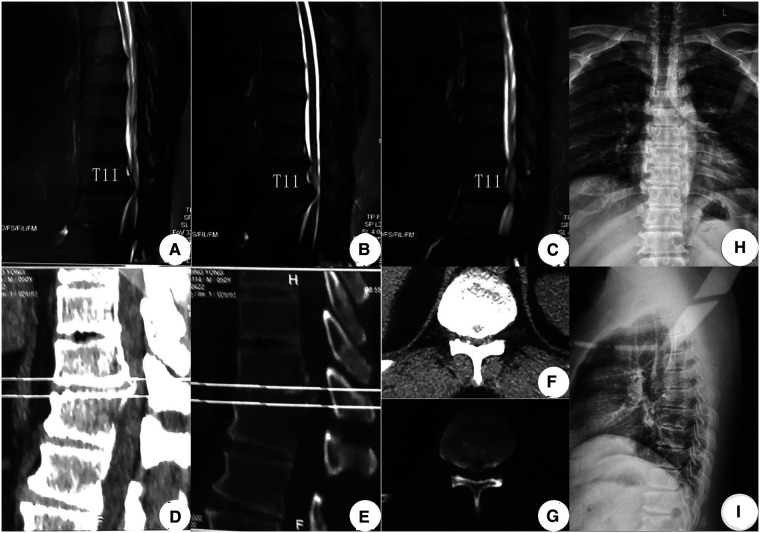
Preoperative image examinations (**A–G**). MR examination done on October 25, 2019, showed a giant TDH at the T11–12 levels: (**A**) Right sagittal T2WI, (**B**) middle sagittal T2WI, and (**C**) left sagittal T2WI. (**D–G**) CT examination recorded on November 14, 2019, confirmed CCTDH at T11/12: sagittal CT images [(**D**) soft tissue window; (**E**) bone window]. Transverse CT images [(**F**) soft tissue window; (**G**) bone window]. (**H,I**) Digital radiograph obtained on November 20, 2019, showed a normal thoracic sequence with no scoliosis. TDH, thoracic disc herniation; T2WI, T2-weighted image; CCTDH, central calcified TDH.

An MR examination done on the same consulting day revealed a giant central TDH that severely compressed the spinal cord from the ventral side at the T11–12 level. Further, a CT examination done on November 14, almost 1 month later, revealed that the central TDH was calcified, following which the patient was formally diagnosed with CCTDH (Figure 1). On the day of admission, he could move his legs but was unable to walk by himself and had a slight difficulty with micturition. The total preoperative modified Japanese Orthopedic Association (mJOA) score was 12 (27). He was admitted to our hospital for decompression surgery on November 20, 2019, after a digital radiograph of the thoracic spinal region was obtained.

## Methods

**Figure 2:**
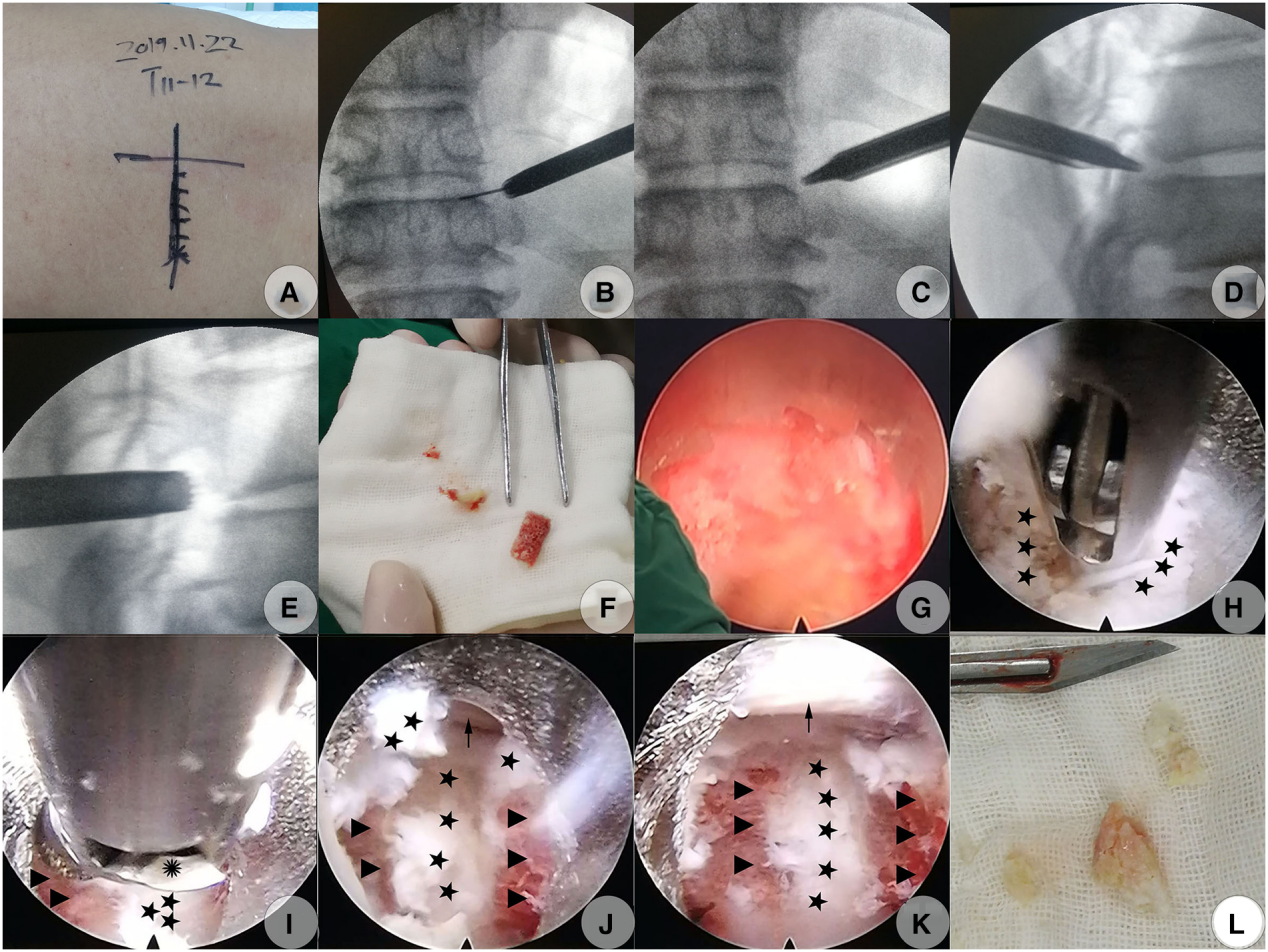
Modified PTES (PTED) to decompress CCTDH on November 22, 2019 (**A–I**). (**A)** Skin mark of the index disc's center guided under C-arm fluoroscopy and skin entrance point determination (Gu's point, the point on the marked transverse line where the flat back turns to the lateral side). (**B**) Subsequently, dilator cannulas were advanced to the anterolateral side of the SAP over the guidewire, which was finally confirmed under AP C-arm fluoroscopy. (**C**) The protection cannula was inserted over the guide rod (which had replaced the dilator cannulas) to kiss the anterolateral side of the SAP which was confirmed under AP C-arm fluoroscopy. (**D**) The beveled end of the protection cannula touched closely to the anterior side of the SAP, which was confirmed under lateral C-arm fluoroscopy. (**E**) The hand trephine firmly caught the lateral portion of the ventral bone from the SAP at the fluoroscopic foraminoplasty step. (**F**) The bone chunk isolated from the ventral portion of the SAP by the trephine. (**G**) The trephine's saw teeth under endoscopic visualization when performing endoscopic foraminoplasty. (**H**) Soft herniated disc fragments ventral to the calcified shell were removed using forceps. (**I**) Calcified shell was thinned using an electric diamond drill at the flexible position. (**J**) Ventral surface of the dural sac on the left side was exposed after the removal of the bony disc shell. (**K**) Dural sac with visible pulsation following complete decompression. (**L**) Isolated disc fragments with scattered calcification. Pentagram, soft fragments of the herniated disc; Triangle, calcified disc shell; Polyhedrosis, hinge of an electric diamond drill; Arrow, dural sac. PTED, percutaneous transforaminal endoscopic decompression; PTES, posterolateral transforaminal endoscopic surgery; CCTDH, central calcified TDH; AP, anteroposterior; SAP, superior articular process.

A modified PTES technique was introduced to perform the decompression procedure (18). The patient was placed in a prone position on a radiolucent table. The target disc was identified under the C-arm. Gu's point (the point on the marked transverse line bisecting the index disc space where the flat back turns to the lateral side) was chosen as the skin entrance point on the left side (we measured the distance between Gu's point and the midline and found that it was 6 cm) (Figure 2A). Following the administration of local anesthesia with 0.5% lidocaine under conscious sedation, which allowed continuous feedback to be obtained from the patient, a soft tissue trajectory was established according to Gu's description (18). Foraminoplasty was performed using a hand trephine, and the procedure was divided into early and later stages. The early-stage foraminoplasty was performed purely under C-arm guidance using a 7.5 mm diameter trephine, which we called fluoroscopic foraminoplasty; the later-stage foraminoplasty was performed under full endoscopic visualization, which we termed endoscopic foraminoplasty.

Fluoroscopic foraminoplasty was done as follows: following the establishment of the soft tissue trajectory (Figure 2B), the dilating cannula was removed and replaced by a 6.3 mm guiding rod (GD) over the guiding wire till the GD's tip touched the lateral edge of the superior articular process (SAP); the GD was then slid into the foramen but with a depth less of than 1.0 cm, touching closely against the anterior edge of the SAP after the removal of the guiding wire. An 8.8-mm outer diameter beveled cannula (protection cannula) was pushed over the GD to dock at the anterolateral edge of the SAP (Figures 2C,D). After the GD was removed, the protection cannula was pressed down slightly horizontally to cover more portions of the ventral aspect of the SAP, with its beveled end touching the ventral aspect of the SAP closely (the so-called pressdown enlargement of the foramen). A hand trephine was then introduced into the protection cannula's cavity to touch the lateral portion of the ventral bone from the SAP firmly. At this point, a forceps was introduced into the reamer's cavity to ensure that a major part of the cavity in the posterior direction was occupied by the bone structure from the SAP, while leaving little free space from the foramen to ensure that the anterior edge of SAP was contained within the reamer's cavity. Then, the trephine was rotated into the SAP with a depth of 5–8 mm to ensure that the trephine’s saw teeth penetrated into the lateral portion of the ventral SAP firmly, while keeping the medial cortex of the SAP intact. Then, a posteroanterior and lateral C-arm view was imaged to identify the direction and location of the trephine. This marked the culmination of fluoroscopic foraminoplasty (Figure 2E).

The later-stage foraminoplasty was done under full endoscopic visualization: A 30° endoscope was inserted *via* the trephine's cavity; the soft tissues adhering to the lateral edge of the SAP that obstructed the observation or confirmation of relative bony landmarks were stripped off just about enough to identify the bony structures under endoscopic visualization with constant saline irritation. The trephine was rotated deep into the desired direction determined by previous C-arm identification till the bone inside the trephine's cavity began to rotate at the same pace with the reamer. Meanwhile, the bony resistance had faded, indicating that the medial cortex could be penetrated, the bone chunk inside the reamer's cavity could be isolated completely from the residual SAP, and the isolated bone chunk could be extracted along with the trephine (Figure 2F). Additional ventral bone from the SAP was further resected as desired in the same fashion to achieve adequate enlargement of the foramen (Figure 2G). The new isolated bones became smaller and could be removed directly with a forceps, while keeping the trephine in the protection canal. Finally, ligamentous flavum lateral to the dural sac and CCTDH was adequately exposed, which signaled the completion of endoscopic foraminoplasty (18).

**Figure 3:**
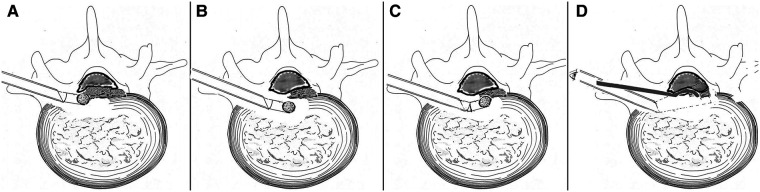
Illustration of the manipulation of a flexible power drill (**A–D**). (**A**) Calcified disc on the left side was removed using a power diamond drill, while the control handle was in the straight position. (**B**) Control handle approached anteromedially in a straight position avoiding dural sac disturbance. (**C**) The distal end of the control handle angled dorsally to remove the calcified shell on the right side (the no-touch technique). (**D**) Endoscope-assisted resection of CCTDH using a curved dissector from another trajectory (not performed in our patient). CCTDH, central calcified TDH.

Then, decompression under endoscopic visualization was started: The trephine and the protection cannula were replaced by a working cannula, and the endoscope was inserted into the working cannula. The inside-out technique was performed initially, that is, decompression *via* the intradisc instead of the intracanal (3). The soft disc fragments ventral to the calcified disc shell was undermined with straight or curved microforceps to form a cavity (Figure 2H). Then, a flexible endoscopic diamond burr (Chongqing Xishan Technology Co., Ltd, Chongqing, China) was inserted to thin the calcified shell (Figure 2I). The direction of the diamond burr was changeable as desired through a hinge at the control handle's distal end, which was linked to the rotatory control button at the handle's proximal end. The maximal flexible angle was 30°. The highest rotation speed was 25,000 r/min. The mechanism of the burr was the same as described by Zhang et al. (25) (Figure 3). The no-touch technique was used to ensure that the fragile dural sac was shielded from even minor disturbance (4). When the bony shell was thinned to a point where it became semitransparent or totally transparent, the diamond drill was removed, and a curved dissector or a flexible radiofrequency probe was used to dissect the thin bony shell from the dura cautiously. The caudal and cephalic bony protrusions were also grinded off by rotating the diamond drill up and down. The combination of the displacement of the working cannula and endoscope in various directions and depths, coupled with the angled endoscopic view with the rotation of the endoscope, fix-angled instruments, and flexible power drill, termed the “joystick technique”, provided sufficient decompression area in all directions. Eventually, the shell was fractured within the cavity piece by piece. It was intraoperatively found that not all dorsal surfaces were occupied by the calcified shell. Some part of the central region was still occupied by soft fragments but containing scattered calcified tissue. These fragments were also removed with microforceps. The patient felt a progressive relief of his legs during the removal of the fragments, initially on the left side and slowly spreading to the right side intraoperatively. Finally, a space could be seen underneath the dura sac, and the dural sac with visible pulsation could be seen, which indicated sufficient decompression (Figures 2J,K). Intraoperative bleeding was minimal and almost negligible, and no drainage was used.

**Figure 4:**
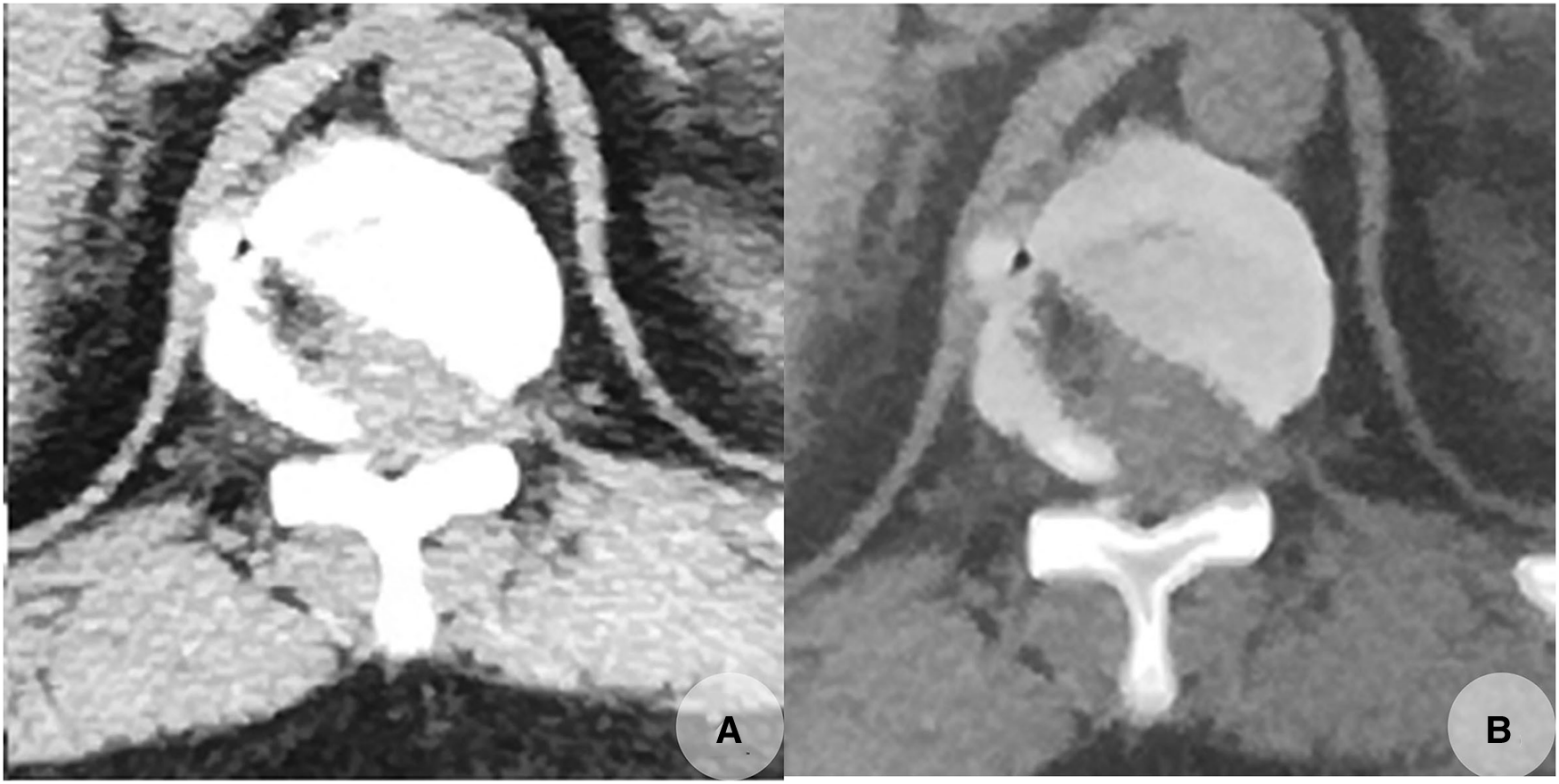
Postoperative CT scans taken on November 23, 2019, revealed that the calcified herniated disc was completely removed with some normal bone excessively removed (**A,B**). (**A,B**) Transverse CT images of T11/12.

The operation time was 100 min. No antibiotics or pain-killer drugs were administrated postoperatively. The patient could walk with a cane 2 days later. The feeling of numbness in his bilateral legs gave way to a welcome relief of tightness. The mJOA score increased to 13. The patient gradually regained a better control of his lower limbs with improved gait and increased muscle strength. A CT re-examination a few days later revealed a complete removal of the herniated disc. The posterior portion of the normal bone structure of the inferior T11 level and the superior T12 level on the contralateral side were unintentionally excessively removed, but thankfully, this did not affect spinal stability (Figure 4).

At 3-month’ follow-up, the muscle strength in both legs returned to almost normal. The patient experienced slight instability but could walk back and forth smoothly unaided; also he had normal micturition. His mJOA score improved to 17. At a 2-year telephonic follow-up, he stated that he could walk freely without any discomfort. The mJOA score improved to 18.

## Discussion

Surgery for CCTDH in the spine is performed extremely rarely, and both its incidence rate and its percentage are very low (1, 2, 3, 6). Traditional approaches have been questioned because of the possibility of a high risk of serious complications, high in-hospital morbidity, and mortality rates, especially when the anterior approaches were chosen (9, 28). The overall trend in the spine surgery technique has been toward less invasiveness, while maintaining similar or better effectiveness and safety (1, 3). PTED has been used to treat noncalcified TDH (1, 2). The central noncalcified disc could be resected using curved microforceps under the angled endoscopic vision (1). Ruetten et al. performed the full-endoscopic uniportal extraforaminal technique under general anesthesia in 15 patients with TDH with calcified intraspinal disc herniations (15). Li et al. performed PTED for soft and paracentral CTDH (22). Paolini et al. reported the use of an endoscope-assisted procedure of CCTDH with a curved dissector from a second trajectory (4). Zhang et al. performed the whole foraminoplasty procedure under endoscopic visualization with a flexible power burr, which we thought would require some time to strip off the soft tissue adhered to the lateral cortex of the SAP, which obstructed the identification of bony landmarks. Meanwhile, to remove the ventral part of the SAP using an endoscopic power burr was considered less fruitful as well as involving a potential displacement of the working cannula, which might result in the surgeon losing sight of the location (25). Compared with a power burr, a hand trephine could help achieve bone removal very quickly.

The original PTES was limited to dealing with lumbar disc herniation (20) including the calcified type successfully (29). We found in our practice that the technique could be expanded to deal with pathology in the lower thoracic spinal region. In our patient, we used the modified PTES technique under local anesthesia, reaping the full advantages of the original PTES technique such as simple orientation, easy puncture, reduced steps, and little x-ray exposure. During the performance of our foraminoplasty procedure, we used a hand trephine instead of a cannulated TomShidi needle and sequential trephines, as the latter was open to frequent x-ray exposure and therefore entailed heightened risk (26).

Our modified technique divided foraminoplasty into fluoroscopic and endoscopic stages. Fluoroscopic foraminoplasty saved precious time as only an insertion of the trephine into the lateral portion of the ventral bone from the SAP was required with an almost precise direction and depth under C-arm confirmation. Endoscopic foraminoplasty ensured safe and adequate foramen enlargement as it prevented iatrogenic damage to the neural structures in the spinal canal, which may cause a puncture of the dura or injury to the traversing nerve. Also, x-ray exposure was reduced. In the endoscopic decompression process, different from the original PTES technique, an inside-out technique was introduced initially, where the cannula is first inserted into the disc just underneath the calcified shell to form a cavity. In the light of the use of the flexible power drill, decompression was performed under full-endoscopic visualization with constant saline irritation. The noncalcified tissues underneath the calcified shell were first removed and then the calcified shell was thinned using the diamond burr. The no-touch technique ensured the safety of the spinal cord by taking into account the patient's feedback under local anesthesia. Thus, by using an endoscopic diamond burr, a modified PTES may prove to be an alternative for CCTDH treatment. It is worth mentioning here that during the follow-up, the patient's condition saw satisfactory improvement, which indicated that our preoperative plan was successful.

However, it should be recognized that this procedure is technically demanding and invariably associated with learning curves. Therefore, it must be performed with utmost care, with the awareness and realization that some normal bone is being excessively removed somewhere intraoperatively (Figure 4).

## Data Availability

The original contributions presented in the study are included in the article/Supplementary Material, further inquiries can be directed to the corresponding authors.
